# Fetal and Placental Weight in Pre-Gestational Maternal Obesity (PGMO) vs. Excessive Gestational Weight Gain (EGWG)—A Preliminary Approach to the Perinatal Outcomes in Diet-Controlled Gestational Diabetes Mellitus

**DOI:** 10.3390/jcm9113530

**Published:** 2020-10-31

**Authors:** Mariusz Gujski, Dariusz Szukiewicz, Marta Chołuj, Włodzimierz Sawicki, Iwona Bojar

**Affiliations:** 1 Department of Prevention of Environmental Hazards and Allergology, Medical University of Warsaw, 02-091 Warsaw, Poland; mariusz.gujski@wum.edu.pl; 2 Chair and Department of General and Experimental Pathology with Centre for Preclinical Research and Technology, Medical University of Warsaw, ul. Pawinskiego 3C, 02-106 Warsaw, Poland; 3 Chair and Department of Obstetrics, Gynecology and Oncology, Medical University of Warsaw, 03-242 Warsaw, Poland; marta.choluj@wum.edu.pl (M.C.); wlodzimierz.sawicki@wum.edu.pl (W.S.); 4 Department of Women’s Health, Institute of Rural Health in Lublin, 20-950 Lublin, Poland; iwonabojar75@gmail.com

**Keywords:** pre-gestational maternal obesity, excessive gestational weight gain, gestational diabetes mellitus, perinatal outcomes, fetal/placental weight ratio

## Abstract

Both pre-gestational maternal obesity (PGMO) and excessive gestational weight gain (EGWG) increase the risk of gestational diabetes mellitus (GDM). Here, we conducted a retrospective study to comparatively examine the relation between fetal birth weight (FW) and placental weight (PW) in PGMO (*n* = 100) compared to EGWG (*n* = 100) with respect to perinatal outcomes in diet-controlled GDM. The control group was made up of 100 healthy pregnancies. The mean FW and the mean PW in EGWG were correlated with lowered fetal weight/placental weight ratio (FW/PW ratio). The percentage of births completed by cesarean section accounted for 47%, 32%, and 18% of all deliveries (EGWG, PGMO, and controls, respectively), with the predominance of FW-related indications for cesarean section. Extended postpartum hospital stays due to neonate were more frequent in EGWG, especially due to neonatal jaundice (*p* < 0.05). The results indicate the higher perinatal risk in mothers with EGWG compared to PGMO during GDM-complicated pregnancy. Further in-depth comparative studies involving larger patient pools are needed to validate these findings, the intent of which is to formulate guidelines for GDM patients in respect to management of PGMO and EGWG.

## 1. Introduction

The prevalence of obesity, defined as a body mass index (BMI) ≥ 30 kg/m^2^, has been increasing worldwide on a pandemic scale with accompanying syndemic health problems [1,2]. These problems currently affect approximately one-third of women of reproductive age and, as a consequence, become more and more widespread during pregnancy [3]. It was proven that obesity increases the risk of both infertility and complicated pregnancy [4,5]. Thus, being closely related to perinatal outcomes, the mother’s obesity can affect maternal and newborn health, posing a major challenge for healthcare providers [5,6,7]. It was thoroughly documented by independent authors that gestational diabetes mellitus (GDM), pregnancy-induced hypertension (PIH), emergency cesarean section, postpartum hemorrhage, wound infections, preterm delivery, large for gestational age (LGA), fetal macrosomia, and intrauterine fetal death (IFD) rates are significantly increased in maternal obesity [8]. Moreover, these obstetrical complications are accompanied by higher neonatal morbidity and mortality [9,10].

In contrast to pre-gestational maternal obesity (PGMO), some mothers experience excessive weight gain during pregnancy [11]. Maternal weight gain in pregnancy can offer a good means of assessing the well-being of the pregnant mother and, by inference, of her baby [12]. Gestational weight gain (GWG) is defined as the amount of weight gained between conception and just before the birth of the infant. Pre-pregnancy BMI is used to assess excessive gestational weight gain (EGWG), as defined in widely accepted published guidelines and recommendations.

In 1990, the Institute of Medicine (IOM, currently known as the National Academy of Medicine) developed recommendations regarding GWG; these guidelines were meant to benefit clinical practitioners. In 2009, these recommendations were updated and incorporated into the World Health Organization’s (WHO’s) definitions for maternal BMI (Table 1) [13]. However, the association between GWG consistent with the IOM guidelines and pregnancy outcomes is unclear [14]. Gestational weight gain recommendations are often exceeded in those with high pre-pregnancy weight (BMI ≥ 30 kg/m^2^), given the narrow range of acceptable GWG recommended in obesity compared to the lower BMI categories (Table 1) [15].

Unfortunately, as is the case for PGMO, EGWG is associated with an increased incidence of maternal and neonatal complications, including hypertensive disorders of pregnancy, fetal macrosomia, and increased cesarean birth rates [11,16,17]. The incidence of neonatal complications is closely related to glucose tolerance during pregnancy. Compared to pregnant women with normal glycated hemoglobin (HbA1c) levels, those with early elevated HbA1c levels were more likely to develop adverse neonatal events, including respiratory distress syndrome (RDS), pneumonia, and jaundice [18]. Moreover, being positively correlated with GDM, the mother’s BMI ≥ 30 kg/m^2^ and EGWG are both associated with an increased risk of childhood overweight/obesity, with the strongest effects at later ages [19,20]. It was recently suggested that even late-pregnancy dysglycemia in obese pregnancies after negative testing for GDM increases the risk of future childhood overweight [21].

GDM is the most common metabolic and endocrine perinatal complication [22]. In GDM, carbohydrate intolerance of variable severity is diagnosed for the first time during pregnancy.

The prevalence of GDM is increasing due to delayed motherhood, the rising prevalence of obesity, and unhealthy lifestyles. However, prevalence estimates produce results that vary widely from 1.8% to 31.5%. This significant variance may be partially explained by reliance on criteria from differing guideline/recommendation sources. Another factor to consider is demographic differences between populations and ethnicities, including how these characteristics affect pregnancy and GDM prevalence [22,23]. According to the most recent (2017) International Diabetes Federation (IDF) estimates, GDM affects approximately 14% of pregnancies worldwide, representing approximately 18 million births annually [24].

The underlying pathophysiology of GDM has not yet been clearly explained; the particular molecular mechanisms underlying GDM remain poorly defined. What we do know, however, is that three central features of pregnancies complicated by GDM include insulin resistance with β-cell dysfunction, low-grade inflammation, and endothelial cell dysfunction [25,26]. These elements are influenced by the amount of adipose tissue present before and/or during the pregnancy [27,28]. Among women with GDM, only 15% will require insulin. Thus, nutritional management is the main treatment for GDM, and overweight/obesity is the principal challenge in patient counseling and interventions during pregnancy [29]. However, despite the fact that both PGMO and EGWG are positively correlated with GDM, exceeding a certain level of metabolic disorders resulting in GDM does not require increase in the adipose tissue mass [30,31,32]. Nevertheless, it is reasonable to check which modifiable risk factor (i.e., PGMO or EGWG) shows the stronger correlation with the fetal and placental weight parameters and some perinatal outcomes recorded after the onset of GDM.

While looking for a simple model to assess birth weight and placental mass in relation to the course of pregnancy and perinatal outcomes, we took into account that, in the healthy population delivered at term, placental weight scales to birth weight to the 3/4 power [33]. This fact may suggest that placental weight is a justifiable proxy for fetal metabolic rate when other measures of fetal metabolic rate are not available. Moreover, independent authors reported the existence of various fetal-placental allometric scaling interrelationships different from a simple dimensional proportionality, with modifying factors that include metabolic imbalances observed in GDM [33,34,35].

The aim of this study was to comparatively examine the relation between fetal birth weight and placental weight in pre-gestational obesity (PGMO) vs. excessive gestational weight gain (EGWG) with respect to selected perinatal outcomes in diet-controlled gestational diabetes mellitus (GDM). 

## 2. Material and Methods

This preliminary retrospective study is based on a review of medical records of patients giving birth in the years 2015 through 2019 at the Department of Obstetrics, Gynecology and Gynecologic Oncology, Faculty of Medicine, Medical University of Warsaw, Poland. The study was conducted in compliance with international and local laws and the research protocol was approved by the local Ethics Committee of the Medical University of Warsaw (ethical clearance approval number KB17/2014).

According to the inclusion/exclusion criteria given in Table 2, group I (PGMO; *n* = 100) and group II (EGWG; *n* = 100) were established. These criteria have been carefully selected to ensure optimal homogeneity in the groups, avoiding as much as possible discrepancies caused by unpredictable or non-detected factors. Considering that placental weight and the fetal weight/placental weight ratio (FW/PW ratio) are also affected by fetal sex, the male/female ratios were similar within the studied groups and amounted to 51/49, 53/47, and 50/50 (group I, group II, and healthy controls, respectively) [36]. All patients were white and all lived in lowland areas in Europe.

The diagnosis of GDM in the PGMO and EGWG groups was based on clinician interpretation of the results of standard glucose tolerance testing during pregnancy, according to recommendations from 2013 WHO publications [38,39]. A two-hour, 75 g oral glucose tolerance test (OGTT) was performed between 24 and 28 weeks of gestation. Patients with GDM stayed under a doctor’s care until delivery, being monitored at consecutive visits in the diabetes outpatient clinic. Nutritional therapy alone was effective to control glucose level within the recommended range. The “upper boundary” treatment targets were to maintain maternal capillary glucose concentrations at <99 mg/dL (<5.5 mmol/L) in the fasting state, <140 mg/dL (<7.8 mmol/L) at one hour, and <127 mg/dL (<7.1 mmoL/L) two hours after starting the meal [41]. These patients determined their glucose levels four times daily with a glucometer. Dietary modifications included reduction of caloric intake for PGMO patients with limitation of carbohydrate content to 35% to 40% of total calories and emphasis on complex rather than simple carbohydrates. In the vast majority of PGMO cases, these nutritional principles were implemented under the guidance and control of a nutritionist experienced in dietary management of women with diabetes mellitus in pregnancy. All patients declared adherence to the dietary treatment, although it is worth noting that it is almost impossible to verify dietary self-reporting.

Glucose control was satisfactory in all GDM patients, as it was confirmed by measurements of HbA1c in the third trimester (Inclusion Criteria in Table 2) [37]. The control group comprised 100 healthy patients with normal BMI and GWG values within the recommended range (11.5 to 16 kg, Table 1), in whom the course of pregnancy was physiological, and ended at term by spontaneous delivery or non-elective cesarean section [13,41]. This group served as a reference when comparing groups I and II.

Maternal-perinatal outcomes included the mother’s total weight gain (kg, rounded to the nearest half), birth weight (g, rounded to the nearest 5 g), fetal/placental weight ratio, mode of delivery (spontaneous or induced delivery, including indications for cesarean section), duration of the second stage of labor, anesthetic management of labor concerning Apgar score (<8 points at 1 min), postpartum maternal and fetal complications, and average hospital stay (days from admission to delivery and from delivery to discharge).

Pregnant women were weighed at regular intervals, including the initial visit at the time of pregnancy confirmation (at approximately 6 weeks of gestation) and pre-delivery week. Following delivery, newborns in a stable state were weighed within 30 min of birth. After severing the umbilical cord, placentas were weighed together with the membranes immediately after delivery in a standardized manner. Gestational age and delivery date were estimated using the last menstrual period (LMP) solely or together with ultrasonography performed between gestational weeks 11 and 14.

The chi-square statistical test was used for identifying relationships between categorical variables. To check whether the data are normally distributed, histograms and the Kolmogorov-Smirnov test were applied. Post hoc Student’s *t* tests or post hoc Mann-Whitney *U* tests were used for pairwise comparisons. A *p* value of <0.05 was considered statistically significant.

## 3. Results

Results pertaining to the analyzed parameters are summarized in Table 3. The mean weight parameter values are presented together with the medians, which shows that distribution of variables was close to normal or strived for normal.

A statistically significant (*p* < 0.05) increase in body weight throughout pregnancy was observed in group II (EGWG) at a higher rate than in group I (PGMO) and the controls, whereas the comparison between group I and the control group was not significant. This outcome indicates that obese women (PGMO) under dietary control achieved similar GWG as pregnant women with normal BMI in the control group (15.2 kg ± SEM 0.97 and 14 kg ± 0.89, respectively). However, these results show that dietary control was suboptimal. According to the National Academy of Medicine (formerly, IOM) guidelines for patients categorized as pre-pregnancy obese (BMI ≥ 30.0), a maximum of 9 kg weight gain during pregnancy is recommended [12] (Table 1). The greatest mean change between “pre-pregnancy” and “at time of delivery” BMI values was observed in EGWG (+ 6.8), whereas in PGMO and control groups the differences amounted to +5.2 and +4.7, respectively.

Significant differences between the groups were observed in relation to birth weight, where, based on the median, value distribution was similar to normal (Table 3). 

The mean gestational ages at the time of delivery were 38 5/7, 39 0/7, and 39 1/7 weeks (groups: PGMO, EGWG, and control, respectively). The sexes of the newborns (male: female ratio) in the studied groups were as follows: 49:50, 51:49, and 52:48 (PGMO, EGWG, and control, respectively).

The highest mean birth weight amounted to 3.815 g ± SEM 191.1 and was recorded in GDM-complicated pregnancy with EGWG (group II). In the PGMO group, the newborn average birth weight was also higher than that of the control group (3.507.5 g ± 207.5 vs. 3.376 g ± 179.9; *p* < 0.05) but significantly lower than in group II. The incidence of fetal macrosomia reflected the differences in birth weight between the study groups (Table 3).

Placental weight (the mean and the median values) in the studied groups showed similar relationships as found in the case of birth weight. The maximum mean value of 825.5 ± 44.6 (percentile: 83.2) was noted in group II, whereas in PGMO patients this value was lower (56.5 ± 33.2 (percentile: 51.4); *p* < 0.05), but still significantly higher than in the controls (568.5 ± 31.6 (percentile: 34.1)).

Remembering some restrictions, it can be assumed that fetal weight/placental weight (FW/PW) ratio or, alternatively, placental weight/birth weight ratio, may be treated as a preliminary assessment of placental efficiency in term pregnancies [42,43]. The differences in FW/PW ratio between the groups are shown in Table 3. Both the highest mean birth weight and the highest mean placental weight in EGWG were correlated with a significantly lower FW/PW ratio, suggesting that this allometric growth of the fetus and placenta reflects the degree of metabolic disorders. Along that line of reasoning, the FW/PW ratio in PGMO, although significantly reduced compared to the control group (4.62 ± 0.37 vs. 5.94 ± 0.29), may correspond to a lesser degree of metabolic imbalances in GDM-complicated pregnancy.

Among the analyzed perinatal outcomes, the rate of cesarean sections and length of perinatal hospitalization are noteworthy. Despite a lack of indications for elective cesarean section prior to pregnancy (Table 2, Exclusion criteria), the percentages of births completed in this way accounted for 47% and 32% of all deliveries in group II and I, respectively, compared to only 18% cesarean section deliveries in the controls (Table 3). The statistically significant differences observed between the groups are even more evident (*p* < 0.02) when comparing indications for cesarean section narrowed to those associated with increased birth weight of the fetus (i.e., suspected fetal macrosomia, poor labor progress, and/or imminent uterine rupture). The shares of such indications in the total number of cesarean sections amounted to 56%, 70%, and 33.3% in EGWG, PGMO, and controls, respectively. Duration of perinatal hospitalization showed differences only in the postpartum period. The differences in the incidence achieved statistical significance when the indications for prolonged postpartum hospitalization (>3 days) were limited to those related to the newborn, especially neonatal jaundice. The incidence of extended postpartum hospital stay due to neonate was similar in group I and the control group (total percentage and neonatal jaundice-related percentage, respectively, 20% and 11% vs. 18% and 10%), whereas in the EGWG group, these incidence rates increased (*p* < 0.05) to 24% and 16% (Table 3).

No differences were found for the other analyzed maternal-perinatal outcomes, including duration of the second stage of labor, anesthetic management of labor, below-normal Apgar score (< 8 points at 1 min), postpartum maternal complications, and average hospital stay (excluding extended postpartum hospital stay due to neonate). Since these less-common outcomes were found in almost equal number across all three study groups, the data on them was omitted from final analysis. It is likely these occurrences would be more prevalent in a study group larger than just 100 in number, but the limited numbers in this study proved this data inconclusive.

## 4. Discussion

There is evidence that obesity negatively affects both oocyte quality and developmental competence [44]. Maternal pre-pregnancy BMI, GWG, insulin resistance, inflammation, and glucose, lipid, leptin, and amino acid concentrations have both independent and interacting effects on fetal growth, operating both early and late in pregnancy [45]. All are sensitive to maternal nutrition [46]. Fetal growth demands a coordinated increase in size of the fetus and the placenta throughout pregnancy. Normal metabolic adaptations in the mother’s body are crucial for functioning of the maternal–placental–fetal unit [47]. Regarding glucose tolerance, pregnancy is a period of unique metabolic plasticity during which mild insulin resistance is a physiological adaptation to prioritize fetal growth. To compensate for this adaptation, pancreatic β-cells utilize a variety of adaptive mechanisms, including increasing mass, number, and insulin-secretory capacity to maintain glucose homeostasis [48]. Insufficiency in such a compensation or decompensation leads to GDM, which is typically associated with metabolic disorder phenotypes such as PGMO, EGWG, low-grade inflammation, and insulin resistance. GDM-associated adverse fetal and neonatal outcomes result from the metabolic milieu projected on the fetus via the placental interface in the form of significantly altered placental expression of many factors, including, among others, insulin-like growth factors (IGFs) and glucose transporters (GLUTs) [49,50]. Therefore, it can be considered to be one of the great obstetrical syndromes [51]. Moreover, it was confirmed that women with GDM are at higher risk of developing glucose intolerance and diabetes later in life [52].

Since birth weight depends on placental function, the basis for any reflection on the relationship between birth weight and placental weight is the fact that the fetoplacental ratio is a common proxy for the balance between fetal and placental growth. A pregnancy-equivalent metabolic scaling equation elaborated the complex relationship between placental nutrient transfer and fetal growth, a finding that suggests these factors can be parsed allometrically [53]. Outside the scope of this publication, this finding also suggests flow or fractal theory might be applicable when studying fetal-placental allometric scaling [34,54].

In our study, as in other independent studies, GDM pregnancies differed from non-diabetic controls in increased birth weight, increased placental weight, and lower FW/PW ratio. Essentially, placental weight is similar to fetal weight in that it tends to be heavier in the presence of diabetes, but placental weight gain is more pronounced than fetal weight gain [55]. It has been previously suggested that placental weight partially mediates the effects of pre-pregnancy obesity, GDM, and EWG on fetal growth among term infants [56].

Actually, it is impossible to determine whether placental overweight is the cause or the consequence of fetal overweight. Interestingly, the distinction between PGMO and EGWG made it possible to see how these allometrically evident disorders are more profound in patients with normal initial BMI, who then experienced EGWG. One explanation for this finding may be related to the observed magnitude of weight gain during pregnancy. Recommendations for pregnancy-related weight gain goals published by the National Academy of Medicine, including definition of excessive weight gain, were used in this study (Table 1). Following these recommendations, we found that GWG in group II was an average of 4.3 kg higher than in group I. In keeping with the published recommendations, women with pre-gestational BMI ≥ 30 are expected to gain less pregnancy weight than those whose pre-gestational BMI was in the normal range (data not provided). It can therefore be assumed that, unlike in PGMO, in EGWG, metabolic disorders with insulin resistance at the forefront are revealed almost exclusively during pregnancy [26]. Thus, the course of metabolic imbalance would be more intense, leading to more severe complications manifested as higher average birth weight, more advanced macrosomia, and/or placentomegaly [57,58]. Moreover, GWG may be treated as an independent risk factor for macrosomia in women with transient glucose intolerance but no overt diabetes [59].

Despite the improvement in maternal glycemic control, structural and functional changes of the diabetic placenta at term may occur independently of the type of diabetes. It is well known from numerous studies that placentomegaly in GDM coexists with a wide spectrum of histopathological findings, such as villous fibrinoid necrosis, villous immaturity, abundance of blood vessels within the terminal chorionic villi due to hypoxia-induced hyperplasia (chorangiosis), and ischemic/inflammatory changes [60,61,62]. Further comparative morphological analyses of GDM-complicated PGMO and EGWG are needed to assess the degree of these changes affecting placental function.

In our study, dietary control of glycemia was satisfactory, as confirmed by HbA1c analyses. However, this fact should not be overestimated because placental response to altered glycemia that could have important consequences for the fetus has been reported in pre-diabetic hyperglycemic pregnant women. Even impaired glucose tolerance (IGT) corresponding to mild gestational hyperglycemia was associated with macrosomia and a higher risk of perinatal mortality [63]. Morphologically, the placenta of these women was characterized by an increase in the number of terminal villi and capillaries, presumably as part of a compensatory mechanism to maintain homeostasis at the maternal-fetal interface. It was demonstrated that a change in the placental VEGF/VEGFR expression ratio in mild hyperglycemia may favor angiogenesis in placental tissue and could explain the hypercapillarization of villi [64,65]. It should be pointed out that in our inclusion criteria, we used an acceptable level of HbA1c < 6.0%, whereas the optimal cutoff point of HbA1c related to IGT diagnosed by OGTT was given as 5.6% [37,66]. Thus, the fact that all our GDM patients showed satisfactory dietary control of hyperglycemia probably reduces but does not exclude the risk of placental morphologic and functional disorders. In addition, it also cannot be ruled out that histopathologic placental changes may exist in PGMO and EGWG independently of GDM. Transient hyperglycemic states are commonly observed in excessive caloric intake with or without obesity [67]. Finally, considering the impacts of obesity, IGT, and GDM on placental morphology, inter-individual variations that occur with epigenetic inheritance should also be taken into account [68]. The interpretation difficulties indicated above partially explain our decision not to perform histopathologic examination of the placentas in the studied groups at this preliminary stage.

Obesity during pregnancy and childbirth is associated with labor dystocia leading to instrumental or operative delivery, but the underlying pathophysiological mechanisms remain unclear. An increased percentage of cesarean sections was demonstrated in both examined groups in comparison to the controls, particularly evident in group II, and was positively correlated with birth weight and fetal macrosomia. In addition to this finding, there are data indicating that contractile activity of the uterus may be impaired by both obesity and diabetes [69,70]. The myometrium undergoes dramatic changes in phenotype from early pregnancy until the onset of labor, characterized by an early proliferative phase, an intermediate phase of cellular hypertrophy and matrix elaboration, a third phase in which the cells assume a contractile phenotype, and the final phase in which cells become highly active and committed to labor [71]. The inflammatory background demonstrated in obesity and GDM may significantly influence the growth and remodeling of the myometrium during pregnancy. It was demonstrated in the myometrium of obese pregnant women at term that reduced myocyte density with increased triglyceride content may be responsible for poorer uterine contractility [72]. In diabetes, including GDM, the increase in citrate synthase activity in the myometrium, coexisting with the lower protein content in the myometrium, may reduce or suspend uterine contractions during labor [73].

In developed countries, there is a trend to shorten the length of postpartum hospital stay driven by cost-limiting procedures, hospital bed availability, and a movement toward “humanization” of childbirth [74]. However, shorter hospital stays may be linked with an increase of neonatal readmissions in the first 28 days postpartum, but do not seem to have an effect on the maternal readmissions [75]. Early postnatal hospital discharge generally refers to the postpartum hospital discharge of the mother and newborn within 48 hours [76]. In our study, extended (>3 days) postpartum hospitalization due to neonate was observed in EGWG (group II; *p* < 0.05), whereas the difference between group I and control group was not significant. In all groups, the most common problem causing prolonged hospital stays was neonatal jaundice. Again, the highest incidence of neonatal jaundice was observed in EGWG, while there were no major differences among the other groups. This is somewhat surprising with respect to group I. High unconjugated (indirect) bilirubin levels are frequently seen in infants of diabetic mothers [77]. Although several mechanisms have been studied, the cause of hyperbilirubinemia remains unclear [78]. Polycythemia raises bilirubin levels, resulting in neonatal jaundice. However, severe hyperbilirubinemia can occur even in the absence of polycythemia [79]. In our study, despite good GDM control, neonates of mothers from group II developed jaundice leading to prolonged hospitalization.

It should be noted that patients’ physical activity during pregnancy was not included in this study. The data on effectiveness of physical activity in prevention and management of GDM are contradictory at this time [80]. Finally, to obtain a more complete picture of the differentiation of the assessed parameters between PGMO and EGWG women, the inclusion criteria for PGMO should be supplemented with the requirement: “maximum weight gain during pregnancy not exceeding 9 kg.”

## 5. Conclusions

To the best of our knowledge, this is the first comparative study addressing the allometric relationships between birth weight and placental weight in GDM-complicated PGMO and EGWG in the context of the perinatal outcomes. The manifold placental changes that reveal themselves at the end of pregnancy should be treated as adaptive responses to protect the fetus from diabetes and obesity. The causal role of the placenta, if any, in mediating long-term effects on prenatal development, is an important area of current and planned research [81].

In conclusion, the results of this preliminary study indicate higher perinatal risk in mothers with EGWG compared to PGMO during GDM-complicated pregnancy. This risk is manifested in the form of a significantly increased percentage of cesarean sections in group II (EGWG), which was positively correlated with significantly higher birth weight in group II and coexisted with longer postpartum hospital stay (*p* < 0.05) due to reasons attributable to the newborn, including increased incidence of neonatal jaundice. However, further in-depth study comparing larger groups of patients is needed to validate these results, so that uniform guidelines can be formulated for GDM management of PGMO and EGWG patients.

## Figures and Tables

**Table 1:** Gestational weight gain recommendations.

Pre-Pregnancy BMI Category	Recommended Total Weight Gain ^a^			
	Singleton Pregnancy		Twin Pregnancy	
	kg	lbs	kg	lbs
BMI < 18.5, Underweight	12.5–18.0	28–40	^b^	^b^
BMI: 18.5–24.9, Normal weight	11.5–16.0	25–35	17.0–25.0	37–54
BMI: 25.0–29.9, Overweight	7.0–11.5	15–25	14.0–23.0	31–50
BMI: 30.0–34.9, Obesity ^c^	5.0–9.0	11–20	11.0–19.0	25–42

^a^ Rounded values; ^b^ Available information is insufficient to develop guidelines for underweight women pregnant with twins; ^c^ Due to insufficient research evidence to recommend weight gain for women with pre-pregnancy BMI ≥ 35.0, the range in this category is narrowed: 30.0–34.9. Adaptation based on The Institute of Medicine (The National Academy of Medicine) Guidelines [12].

**Table 2:** Inclusion and exclusion criteria for the respectively studied groups: pre-gestational maternal obesity (PGMO; group I) and excessive gestational weight gain (EGWG; group II).

Inclusion Criteria (Groups I and II)	Exclusion Criteria (Groups I and II)
- Gravidity/Parity: primagravida or primiparous woman after normal pregnancy ended in spontaneous delivery at term - Mother 19–35 years old - Singleton pregnancy - Natural conception, no infertility treatment - GDM diagnosed at 24–28 weeks of the present gestation using OGTT ^a^ - GDM management consisted of diet alone - Glycated hemoglobin (HbA1c) level < 6.0 (42 mmol/mol) [37]; the measurement perfor-med in the third trimester more than 8 weeks from the diagnosis of GDM - Normal thyroid function (euthyroidism) - Delivery at term ^c^ - Vertex presentation of the fetus at the time of labor	- Gravidity/Parity: multigravida or multipara, grand multigravida or grand multi para - Mother’s age: < 19 or > 35 years - Multiple pregnancy - Assisted reproduction (artificial insemination, IVF ^b^, etc.) - GDM diagnosed in the previous pregnancy/pregnancies - The treatment regimen in GDM included not only diet - Glycated hemoglobin (HbA1c) level ≥ 6.0 (42 mmol/mol); the measurement performed in the third trimester more than 8 weeks from the diagnosis of GDM - Hypothyroidism or hyperthyroidism - Preterm or post-term delivery - Pre-existing hypertension including PIH ^d^ in previous pregnancy (if applicable) - Chronic inflammatory conditions - Renal disease - Liver disease - Documented history or presence of arterial or venous thrombosis - Drug or alcohol abuse before/during pregnancy - Cigarette smoking during pregnancy - No reliable data available regarding BMI values - Indications for elective cesarean section prior to pregnancy
**Inclusion Criteria (Group I Only)**	**Exclusion Criteria (Group I Only)**
- Pre-gestational BMI ≥ 30.0 (obesity)	- Pre-gestational BMI < 30.0
**Inclusion Criteria (Group II Only)**	**Exclusion Criteria (Group II Only)**
- Pre-gestational BMI: 18.5–24.9 (normal weight) - Total weight gain > 16.0 kg	- Pre-gestational BMI below or above the range of normal weight (18.5–24.9) - Total weight gain < 16.0 kg (if applicable)

^a^ OGTT—two-hour (75 g) oral glucose tolerance test [38,39]; ^b^ IVF—in vitro fertilization; ^c^ according to “Recommended Classification of Deliveries from 37 Weeks of Gestation” of The American College of Obstetricians and Gynecologists Committee on Obstetric Practice Society for Maternal-Fetal Medicine [40]; ^d^ PIH—pregnancy-induced hypertension; GDM— gestational diabetes mellitus.

**Table 3:** Comparison of selected parameters related to term pregnancy and perinatal period between three groups of pregnant women.

Parameter	PGMO (Group I)	EGWG (Group II)	Control
Mean BMI (kg/m^2^)			
Pre-pregnancy (± SEM)	32.7 (± 2.9) ^♦^	22.3 (± 3.2)	22.8 (± 3.1)
At delivery (± SEM)	37.9 (± 3.3) ^♦^	29.1 (± 3.4)	27.5 (± 3.0)
Total weight gain (kg)			
Mean (± SEM)	15.2 (± 0.97)	19.5 (± 1.01) *	14 (± 0.8)
Median (range)	15 (11–25)	20 (16.5–23.5)	13.5 (11.5–16)
Birth weight (g)			
Mean (± SEM)	3507.5 (± 207.5) ^#^	3815 (± 191.1) *	3376 (± 179.9)
Median (range)	3425 (2710–4760)	3625 (3110–4810)	3372 (2630–4360)
Fetal macrosomia (%) ^‡^	10	11	6
Placental weight (g)			
Mean (± SEM)	656.5 (± 33.2) ^#^	825.5 (± 44.6) *	568.5 (± 31.6)
Median (range)	688 (532–893.5)	797 (614–987.9)	556.5 (453.5–701)
Fetal weight/placental weight ratio (unitless; ± SEM)	5.34 (± 0.3) ^#^	4.62 (± 0.37) *	5.94 (± 0.29)
Cesarean section rate (%)			
Total:			
- Indications:	32 ^#^	47 *	18
A: suspected fetal macrosomia, poor labor progression and/or imminent uterine rupture (% in total)	18 (56) ^#^	33 (70) *	6 (33.3)
B: other non-elective obstetric indications	14	14	12
Mother’s extendent (>3 days)			
postpartum hospital stay due to neonate (%)			
Total:	20	24 *	18
A: neonatal jaundice	11	16 *	10
B: other neonatal-side reasons	9	8	8

PGMO—pre-gestational maternal obesity, EGWG—excessive gestational weight gain (EGWG) and normal control. ^#^ indicates *p* < 0.05 (PGMO versus EGWG); * indicates *p* < 0.05 (EGWG versus PGMO and control); ^♦^ indicates *p* < 0.05 (PGMO versus EGWG and control); **^‡^** fetal macrosomia defined as birth weight over 4000 g irrespective of gestational age.
